# Role of stearyl-coenzyme A desaturase 1 in mediating the effects of palmitic acid on endoplasmic reticulum stress, inflammation, and apoptosis in goose primary hepatocytes

**DOI:** 10.5713/ajas.20.0444

**Published:** 2020-10-14

**Authors:** Bincheng Tang, Jiamin Qiu, Shenqiang Hu, Liang Li, Jiwen Wang

**Affiliations:** 1 Farm Animal Genetic Resources Exploration and Innovation Key Laboratory, Sichuan Agricultural University, Chengdu, Sichuan, 61130, China

**Keywords:** Stearyl-coenzyme A Desaturase 1 (*SCD1*), Goose Primary Hepatocytes, LO2 Cells, Palmitic Acid Tolerance

## Abstract

**Objective:**

Unlike mammals, goose fatty liver shows a strong tolerance to fatty acids without obvious injury. Stearyl-coenzyme A desaturase 1 (SCD1) serves crucial role in desaturation of saturated fatty acids (SAFs), but its role in the SAFs tolerance of goose hepatocytes has not been reported. This study was conducted to explore the role of *SCD1* in regulating palmitic acid (PA) tolerance of goose primary hepatocytes.

**Methods:**

3-(4, 5-dimethylthiazol-2-yl)-2, 5-diphenyltetrazolium bromide was examined to reflect the effect of PA on hepatocytes viability, and quantitative polymerase chain reaction was used to detect the mRNA levels of several genes related to endoplasmic reticulum (ER) stress, inflammation, and apoptosis, and the role of *SCD1* in PA tolerance of goose hepatocytes was explored using RNA interfere.

**Results:**

Our results indicated that goose hepatocytes exhibited a higher tolerant capacity to PA than human hepatic cell line (LO2 cells). In goose primary hepatocytes, the mRNA levels of fatty acid desaturation-related genes (*SCD1* and fatty acid desaturase 2) and fatty acid elongate enzyme-related gene (elongase of very long chain fatty acids 6) were significantly upregulated with 0.6 mM PA treatment. However, in LO2 cells, expression of ER stress-related genes (x box-binding protein, binding immunoglobulin protein, and activating transcription factor 6), inflammatory response-related genes (interleukin-6 [*IL-6*], interleukin-1β [*IL-1β*], and interferon-γ) and apoptosis-related genes (bcl-2-associated X protein, b-cell lymphoma 2, *Caspase-3*, and *Caspase-9*) was significantly enhanced with 0.6 mM PA treatment. Additionally, small interfering RNA (siRNA) mediated downregulation of *SCD1* significantly reduced the PA tolerance of goose primary hepatocytes under the treatment of 0.6 mM PA; meanwhile, the mRNA levels of inflammatory-related genes (*IL-6* and *IL-1β*) and several key genes involved in the phosphoinositide 3-kinase (PI3K)/protein kinase B (AKT), forkhead box O1 (FoxO1), mammalian target of rapamycin and AMPK pathways (*AKT1*, *AKT2*, *FoxO1*, and sirtuin 1), as well as the protein expression of cytochrome C and the apoptosis rate were upregulated.

**Conclusion:**

In conclusion, our data suggested that *SCD1* was involved in enhancing the PA tolerance of goose primary hepatocytes by regulating inflammation- and apoptosis-related genes expression.

## INTRODUCTION

In mammals, excessive fat deposition in hepatocytes causes endoplasmic reticulum (ER) stress, inflammation, and apoptosis, consequently leading the non-alcohol fatty liver to exacerbate into fatty hepatitis [1]. Goose (*Anser anser*), as a descendant of migrant birds, has an excellent capacity to deposit fat in the liver. In poultry production, this capacity is exploited for producing the fatty liver that is 5- to 10-fold larger than normal liver after 2 to 3 weeks of overfeeding [2,3]. Interestingly, their livers can develop serious steatosis without overt injury [4], showing minimal inflammation and other immune-related responses, which suggests that geese have different physiological and metabolic characteristics from mammals. In a previous study, the mRNA level of tumor necrosis factor alpha was significantly downregulated by overfeeding in the goose liver [5]. Moreover, during the formation of goose fatty liver, expression of ER stress marker genes, 78 kDa glucose-regulated protein and X-box binding protein 1, were also inhibited [6]. These results demonstrated that goose liver has a strong tolerance to fatty acids so as to deposit a large amount of fat without the occurrence of lesions.

Stearoyl-CoA desaturase (SCD) is recognized as a pivotal enzyme in the biosynthesis of monounsaturated fatty acids (MUFAs) by catalyzing the insertion of the cis double bond at the delta-9 position of stearoyl-CoA (C16:0) and palmitoyl-CoA (C18:0), which are subsequently converted into corresponding MUFAs, respectively [7]. In the past several decades, studies on *SCD1* were mainly related to fat synthesis, as *SCD1*-knocked mice showed reduced triglyceride (TG) content, inhibited de novo synthesis of fatty acids, and less hepatic steatosis [8]. Several recent reports supported the notion that *SCD1* is critical for regulating the ratio of saturated fatty acids (SFAs) to MUFAs. Janikiewicz et al [9] reported that *SCD1* influenced ER stress response of pancreatic β-cells by altering the SFAs to MUFAs ratio. In another study, Iwai et al [10] found that *SCD1* affected apoptosis in mouse proximal tubular cells by regulating the MUFAs/SFAs ratio. Moreover, it was found that in overfeeding-induced goose fatty liver, the MUFAs/SFAs ratio significantly increased while the content of both palmitic acid (PA) and stearic acids decreased [4,11]. Thus, it can be generalized that *SCD1* exerts an important role in regulating MUFAs/SFAs ratio.

In geese, *SCD1* is ubiquitously expressed in metabolic tissues and shows higher expression levels in liver [1,12]. In addition, goose *SCD1* has higher genomic copy number than that of humans and chickens [13]. Therefore, we hypothesized that *SCD1* could be involved in the regulation of goose hepatic steatosis. To test our hypothesis, this study aims to i) compare the effects of PA at different concentrations on cell viability using 3-(4, 5-dimethylthiazol-2-yl)-2, 5-diphenyltetrazolium bromide (MTT) as well as expression of genes related to ER stress, inflammation, and apoptosis between goose primary hepatocytes and LO2 cells, ii) examine the effects of downregulating *SCD1* expression via RNA interference on cell viability as well as expression of ER stress, inflammation, and apoptosis-related genes in goose hepatocytes under the treatment PA, and iii) investigate the effects of downregulating *SCD1* expression via RNA interference on expression of key genes involvement in phosphoinositide 3-kinase (PI3K)/ protein kinase B (AKT), forkhead box O1 (FoxO1), mammalian target of rapamycin (mTOR) and AMPK pathways in goose hepatocytes under the treatment PA. These data may provide a better understanding of the molecular mechanisms underlying *SCD1* regulating the tolerance of SFAs in goose primary hepatocytes.

## MATERIALS AND METHODS

### Animal care

All experimental protocols involving animal manipulation were approved by the Institutional Animal Care and Use Committee (IACUC) of Sichuan Agricultural University (Permit No. DKY-B20141401).

### Isolation and culture of goose primary hepatocytes

Hepatocytes were isolated from ten 20-day-old Tianfu meat geese (*Anser anser*) that were hatched at the same time and raised under the condition of natural light and temperature at the Experimental Farm for Waterfowl Breeding of Sichuan Agricultural University (Sichuan, China) according to the methods introduced by Seglen [14], and were then cultured with Dulbecco’s modified Eagle’s medium (DMEM; Hyclone, Logan, UT, USA) containing 10% fetal bovine serum (Gibco, Grand Island, NY, USA). The cells were incubated at 37°C in a humidified atmosphere containing 5% CO_2_, and the medium was renewed after 3 h of culture. 24 h later, the medium was replaced with serum-free DMEM medium. After another 12 h, cells were separately treated with DMEM containing 10% fetal bovine serum supplement with 0.2, 0.3, 0.4, 0.5, 0.6, 0.7, 0.8 or 0.9 mM PA and incubated for 24 h, while the control sample cells were cultured with DMEM containing 10% fetal bovine serum media for 24 h. The cells were collected after 24 h of culture. For each experiment, each treatment was repeated in triplicates.

### Culture of LO2 cells

LO2 cells were purchased from Cell Bank of Chinese Academic of Science (Shanghai, China). Cells were cultured at 37°C and 5% CO_2_ in DMEM (Hyclone, USA), supplemented with 10% fetal bovine serum (Gibco, USA). Culture medium was renewed after 3 h followed by the addition of serum-free media after 24 h. After another 12 h, cells were separately treated with DMEM containing 10% fetal bovine serum supplement with 0.2, 0.3, 0.4, 0.5, 0.6, 0.7, 0.8 or 0.9 mM PA and incubated for 24 h, while the control sample cells were culture with DMEM containing 10% fetal bovine serum media for 24 h. The cells were collected after 24 h of culture. For each experiment, each treatment was repeated in triplicates.

### Small interfering RNA transfection

Cultured goose primary hepatocytes were seeded in 6-well plates and incubated for 24 h. Cells were transfected with 0.6 mM small interfering RNA (siRNA) interfere *SCD1*, Albumin from bovine serum or negative control siRNA (GenePharma Co., Ltd, Shanghai, China) using Lipofectamine 3000 transfection reagent (Invitrogen, Carlsbad, CA, USA), and incubated for 24 h with DMEM (Hyclone, USA) containing 10% fetal bovine serum (Gibco, USA). The siRNA sequences are as follows: siRNA-230, GCG AUA CGU CUG GAG GAA UTT (sense) and AUU CCU CCA GAC GUA UCG CTT (antisense); siRNA-602, GCG ACA UAA AGG CCG ACA A (sense) and UUG UCG GCC UUU AUG UCG C (antisense); siRNA-818, CGU ACG AUC AGA ACA UCA A (sense) and UUG AUG UUC UGA UCG UAC G (antisense).

### 3-(4, 5-dimethylthiazol-2-yl)-2, 5-Diphenyltetrazolium bromide assay

The assay for cell viability was performed to Natali et al [15]. Goose primary hepatocytes and LO2 cells were plated at a density of 1×10^4^ cells/well in a 96-well culture dish. After 24 h of PA treatment, cells were then incubated for 4 h with 1 mg/mL MTT in a 37°C incubator, which is converted from the yellow tetrazolium compound to the purple formazan derivative by mitochondria of living cells. After removal of the unconverted MTT, the formazan product was dissolved in dimethyl sulfoxide (DMSO) and the formazan dye absorbance was measured at 490 nm.

### TdT-mediated dUTP Nick-End Labeling (TUNEL) assay

TdT-mediated dUTP Nick-End Labeling (TUNEL) staining was performed with an insitu apoptosis detection kit (Beyotime Biotechnology, Shanghai, China). TUNEL-positive and -negative cells were counted in four randomly selected areas of a culture slide. The results are expressed as a ratio of TUNEL-positive cells to total cell number in each field.

### Cytochrome c immunofluorescence and 4′, 6-diamidino-2-phenylindole assay

The cyt-c protein expression in goose primary hepatocytes was detected by using cytochrome c (cytochrome C antibody) Kit (Beyotime Biotechnology, China) according to the manufacturer’s instruction. 4′, 6-Diamidino-2-phenylindole (DAPI) staining was used with DAPI Staining Solution (Beyotime Biotechnology, China), and cells were incubated for 10 min with 0.5 μg/mL DAPI Solution in a 37°C incubator.

### RNA extraction and cDNA synthesis

Total RNA was extracted from cultures cells using TRIzol Reagent (Invitrogen, USA) according to the manufacturer’s instruction. The quality and quantity of total RNA were checked by electrophoresis on a 1.5% agarose gel. The cDNA was obtained by a cDNA Synthesis Kit (Takara, Shiga, Japan) under the manufacturer’s protocol with 1 μg of total RNA as a template.

### Quantitative real-time polymerase chain reaction

The primers (Table 1) used for quantitative real-time polymerase chain reaction (qRT-PCR) were designed using the Primer Premier 5 software (Premier Biosoft International, Palo Alto, CA, USA). Goose glyceraldehyde-3-phosphate dehydrogenase (*GAPDH*) (GenBank NO. MG674174.1), human-*GAPDH* (GenBank NO. AF261085.1), goose-*β-actin* (GenBank NO. M26111.1), and human- *β-actin* (GenBank NO. DQ407611.1) were used as the internal controls. The mRNA expression levels of target genes were measured by qRT-PCR. The qRT-PCR was performed in a 96-well Bio-Rad iQ5 (Bio-Rad Laboratories, Hercules, CA, USA) using a Takara ExTaq RT-PCR Kit and SYBR Green as the detection dye (Takara, Japan). Real-time PCR was carried out under the following condition: 1 cycle of pre-denaturation at 95°C for 10 s; 40 cycles of 95°C for 5 s, and 60°C for 40 s, and starting at a temperature of 55°C and increasing by 0.5°C every 10 s to determine primer specificity. All cDNA samples were tested three times, and the results were normalized to the levels of *GAPDH* and *β-actin* expression.

### Data analysis

The relative mRNA expression of the target gene was calculated by the comparative Ct method (2^−ΔΔCt^ methods) [16]. Statistical analysis was performed using the SPSS 23.0 software (IBM, Chatsworth, CA, USA). The means of the control and different treatments were subjected to analysis of variance testing, the means were assessed for significance by Tukey’s test, and t-tests were used to analyze the significance between the two groups. Results are presented mean±standard deviation. Differences were considered statistically significant at p<0.05.

## RESULTS

### Comparative analysis of the PA tolerance of goose and human hepatocytes

**Figure 1:**
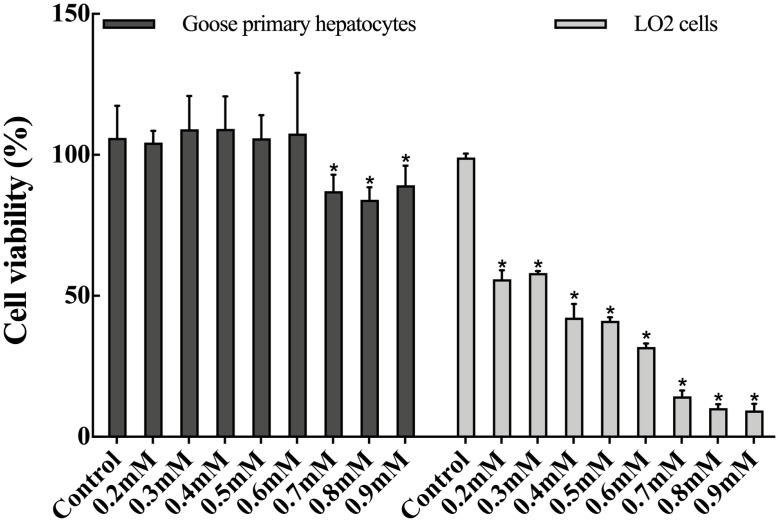
Effects of palmitic acid at different concentrations on cell viability of goose primary hepatocytes and LO2 cells. * Indicates a significant difference at p<0.05 compared to the control group.

The results showed that goose primary hepatocytes had a higher tolerant capacity to PA than LO2 cells (Figure 1). When treated with 0.2 to 0.6 mM PA, the cell viability of goose primary hepatocytes was not changed, but under 0.7 to 0.9 mM treatment, the cell viability significantly decreased (p<0.05). For LO2 cells, when treated with 0.2 to 0.9 mM PA, the cell viability significantly decreased (p<0.05), and with the increase of PA concentration, the cell viability decreased continuously.

### Effects of PA on the mRNA levels of ER stress-, inflammation- and apoptosis-related genes in goose primary hepatocytes and LO2 cells

**Figure 2:**
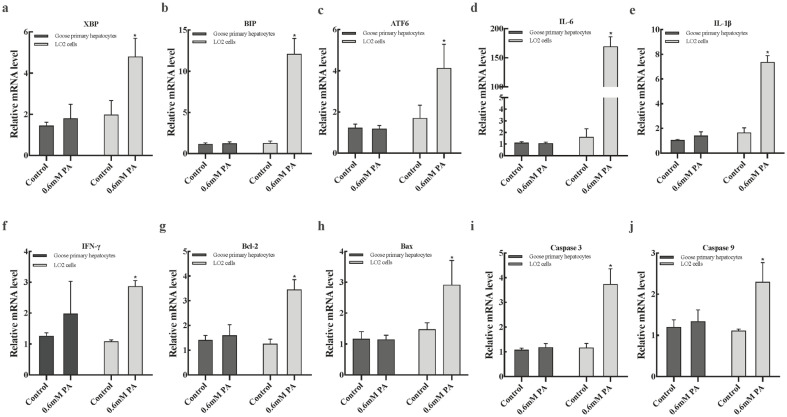
Effects of palmitic acid on (a–c) ER stress-related genes mRNA expression, (d–f) inflammation-related genes mRNA expression, (g–j) apoptosis-related genes mRNA expression in goose primary hepatocytes and LO2 cells. PA, palmitic acid. * Indicates a significant difference at p<0.05 compared to control group of the same species.

To further verify PA tolerance of goose primary hepatocytes, we then compared the mRNA levels of ER stress-, inflammation-, and apoptosis-related genes in both cells. As shown in Figure 2, when treated with 0.6 mM PA, the mRNA levels of ER stress-related genes (x box-binding protein [*XBP*], binding immunoglobulin protein [*BIP*], and activating transcription factor 6 [*ATF6*]), inflammatory response-related genes (interleukin-6 [*IL-6*], interleukin-1β [*IL-1β*], and interferon-γ [*IFN-γ*]) and apoptosis-related genes (bcl-2-associated X protein (*Bax*), b-cell lymphoma 2 (*Bcl-2*), *Caspase-3*, and *Caspase-9*) were not significantly different from the control group in goose primary hepatocytes, but in the LO2 cells their expression levels significantly increased compared to the control group (p<0.05).

### Effects of PA on lipid accumulation related genes expression in goose primary hepatocytes and LO2 cells

**Figure 3:**
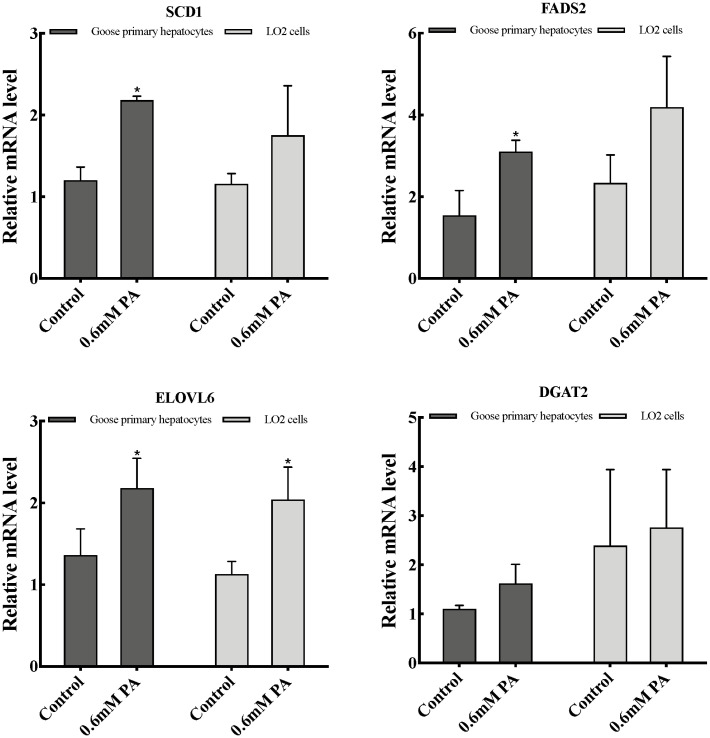
Effects of palmitic acid on TG synthesis related-genes expression in goose primary hepatocytes and LO2 cells. TG, triglyceride; PA, palmitic acid. * Indicates a significant difference at p<0.05.

As shown in Figure 3, the mRNA levels of fatty acid desaturation-related genes (*SCD1* and fatty acid desaturase 2 [*FADS2*]) was significantly promoted (p<0.05) in goose primary hepatocytes, while no significant differences were seen in LO2 cells. In addition, the mRNA expression of the fatty acid elongate enzyme-related gene (elongase of very long chain fatty acids 6 [ELOVL6]) in both cells significantly increased (p<0.05), whereas that of TG synthesis-related gene (diacylglycerol acyltransferase 2 [*DGAT2*]) remained statically unchanged in these two cells.

### Effects of SCD1 downregulation on cell viability and the mRNA levels of ER stress-, inflammation- and apoptosis-related genes in goose primary hepatocytes

**Figure 4:**
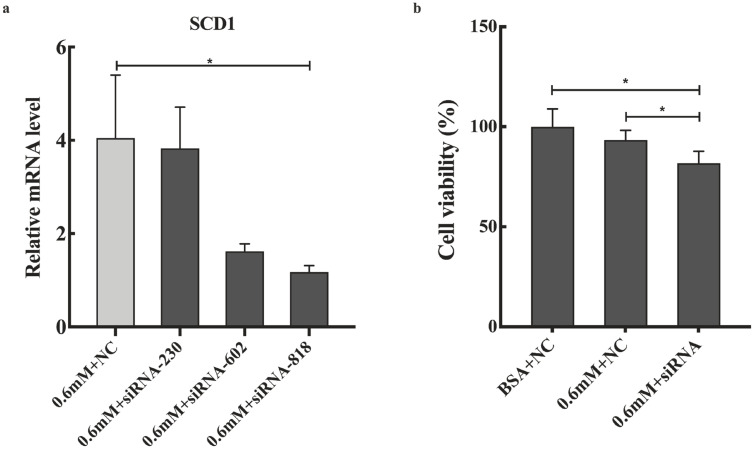
(a) The different siRNA silencing efficiency; (b) Effects of *SCD1* downregulation on goose primary hepatocytes cell viability under palmitic acid treatment. *SCD1*, stearyl-coenzyme A desaturase 1; BSA, albumin from bovine serum. * Indicates a significant difference at p<0.05.

**Figure 5:**
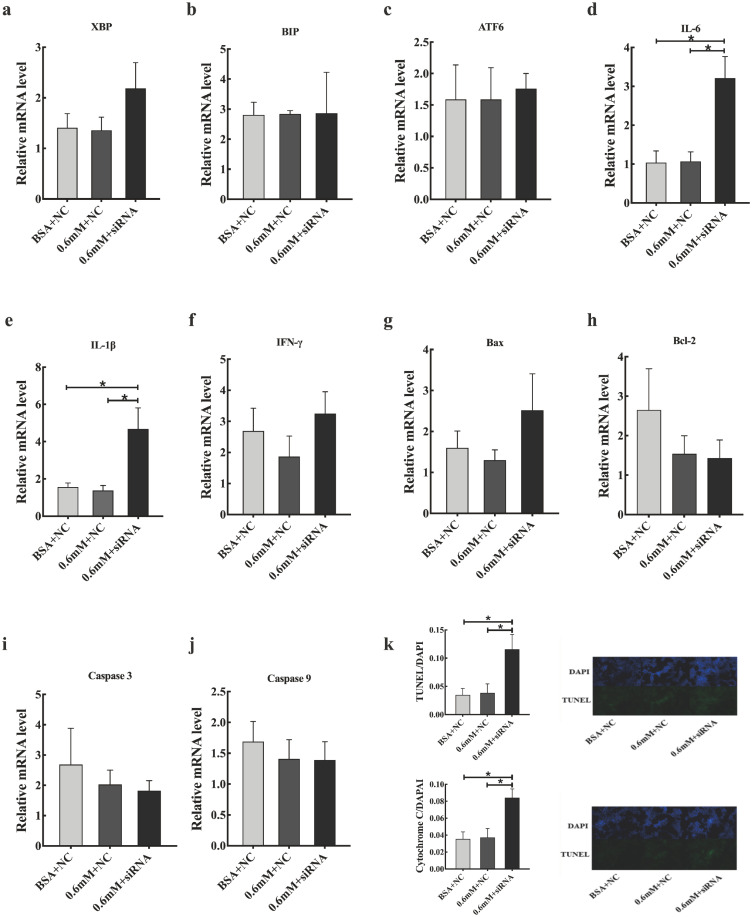
Effects of *SCD1* downregulation on (a–c) the mRNA expression of ER stress-related genes, (d–f) the mRNA expression of inflammation-related genes, (g–j) the mRNA expression of apoptosis-related genes, (k) the apoptosis rate and cyt-c protein expression in goose primary hepatocytes under 0.6 mM palmitic acid treatment. *SCD1*, stearyl-coenzyme A desaturase 1; ER, endoplasmic reticulum; BSA, albumin from bovine serum. * Indicates a significant difference at p<0.05.

The expression profile of *SCD1* promoted us to investigate its function in PA tolerance of goose primary hepatocytes. Then, we interfered *SCD1* in goose primary hepatocytes by using siRNA. As shown in Figure 4, the inhibition efficiency of siRNA-230, siRNA-602, and siRNA-818 were 5.43%, 60%, and 70.86%, respectively. siRNA-818 was the most effective silencing *SCD1* (p<0.05). Therefore, subsequent experiments used siRNA-818 for *SCD1* interference. Under the treatment of 0.6 mM PA, downregulation of *SCD1* significantly reduced the tolerance of goose primary hepatocytes to PA (p<0.05). As shown in Figure 5, under the treatment of 0.6 mM PA, downregulation of *SCD1* significantly increased the mRNA levels of inflammation related genes (*IL-6* and *IL-1β*), the protein expression of cytochrome C and the apoptosis rate. However, genes involved in ER stress (*XBP*, *BIP*, and *ATF6*), inflammatory (*IFN-γ*) and apoptosis (*Bax*, *Bcl-2*, *Caspase 3*, and *Caspase 9*) were not significantly changed under the treatment.

### Effects of SCD1 downregulation on the mRNA levels of PI3K/AKT, FoxO1, mTOR, and AMPK pathway-related genes in goose primary hepatocytes

**Figure 6:**
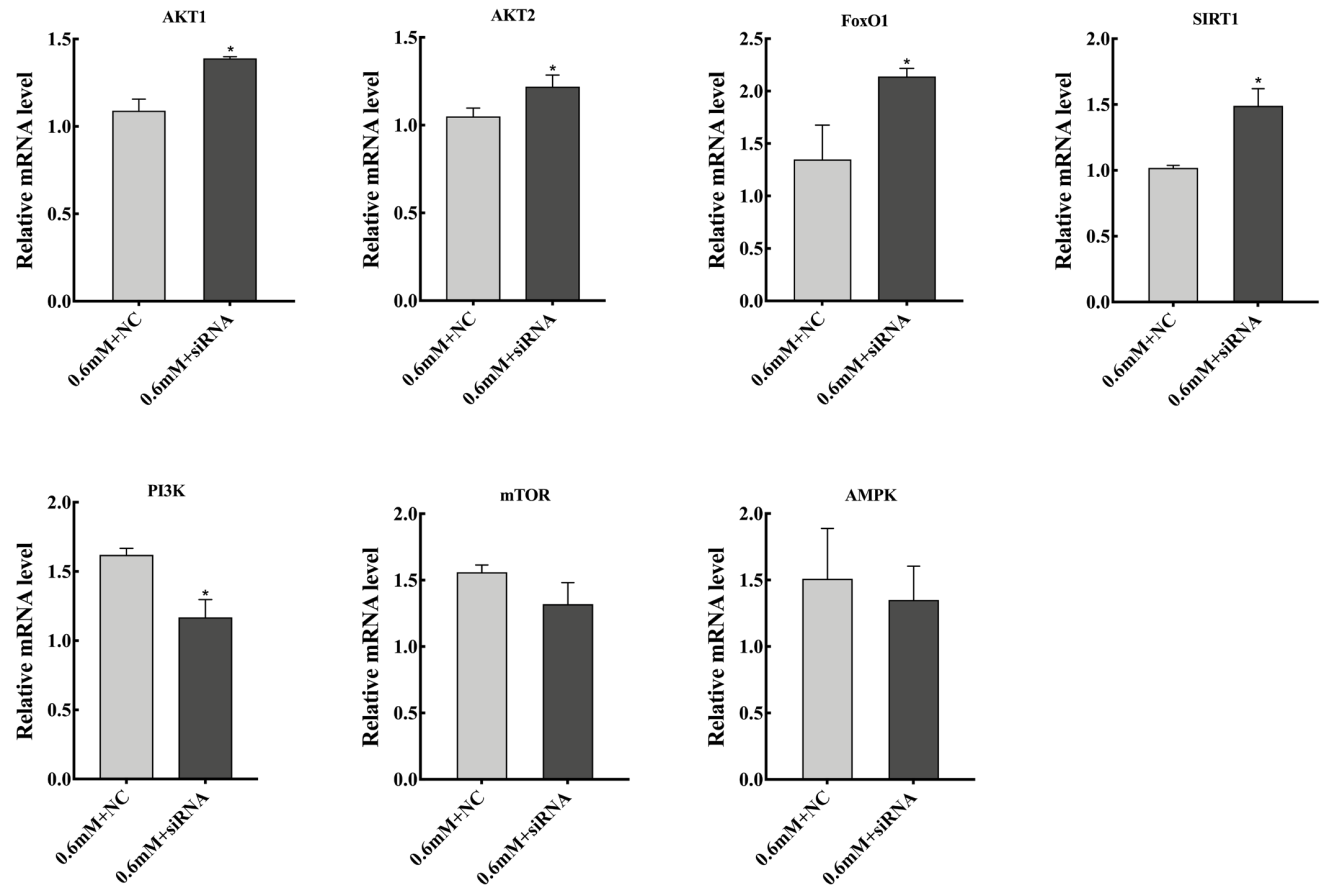
Effects of *SCD1* downregulation on the mRNA levels of PI3K/AKT, FoxO1, mTOR and AMPK pathway-related genes in goose primary hepatocytes under 0.6 mM palmitic acid treatment. *SCD1*, stearyl-coenzyme A desaturase 1; PI3K, phosphoinositide 3-kinase; FoxO1, forkhead box O 1; mTOR, mammalian target of rapamycin. * Indicates a significant difference at p<0.05.

Finally, we detected the expression of *SCD1* regulatory pathway-related genes after downregulating *SCD1*. Under the treatment of 0.6 mM PA, downregulation of *SCD1* in goose primary hepatocytes significantly increased the mRNA expression of pathway key genes (*AKT1*, *AKT2*, *FOXO1*, and sirtuin 1 [*SIRT1*]), and the expression of *PI3K* was decreased (p<0.05), while that of *mTOR* and *AMPK* were not no significantly altered (Figure 6).

## DISCUSSION

PA is a major type of SFAs in the liver and plays essential roles in maintaining liver health. Previous study reported that 0.2 mM PA markedly decreased the cell viability in HepG2 cells [17]; in contrast, our laboratory demonstrated that the cell viability of goose primary hepatocytes was not changed by treatment with 0.6 mM PA [18], suggesting that there may be significant differences in the PA tolerance between goose and human hepatocytes. In this study, the PA of 0.2 to 0.9 mM was used to treat goose primary hepatocytes and LO2 cells cultured *in vitro*. Our results showed that the maximum tolerant concentration to PA of goose primary hepatocytes was 0.6 mM, which was consistent with the results of Pan et al [18]; however, the maximum tolerance concentration of PA in LO2 cells is less than 0.2 mM, which was consistent with the results of the study on HepG2 cells [17]. The above results showed that goose primary hepatocytes have a higher tolerance to PA than LO2 cells, which may be one of the reasons why goose liver has an excellent capacity for fat accumulation.

To fully explore PA tolerance of goose primary hepatocytes, we then compared the mRNA levels of ER stress-, inflammation-, and apoptosis- related genes in goose primary hepatocytes and LO2 cells. ER stress, inflammation, and apoptosis are important bases for evaluating the tolerance of fatty acids in hepatocytes. ER stress occurs extensively in the livers of individuals with steatohepatitis [19]. Previous studies indicated that addition of PA increased the expression of ER stress-related genes in mammalian hepatocytes [20,21]. In this study, we found that treatment with 0.6 mM PA had no significant effect on the mRNA expression of ER stress-related genes (*XBP*, *BIP*, and *ATF6*) in goose primary hepatocytes. However, in LO2 cells, levels of ER stress-related genes were markedly increased (p<0.05), and mRNA levels of *BIP* was increased more than 10-fold. *XBP*, *ATF6*, and *BIP* are located on three pathways that cause the unfolded protein response of ER stress [19], the mRNA expression of the three genes did not change significantly in goose primary hepatocytes, indicating that goose primary hepatocytes have a strong tolerance to PA in ER stress response. In addition, increasing evidence indicated that treatment of mammalian hepatocytes with PA can increase the content of cellular pro-inflammatory factors [22,23]. Inflammatory factors *IL-6*, *IL-1*, and *IFN-γ* are used as biomarkers of hepatitis in mammals, and their expression has significantly increased in mammalian fatty hepatitis. Both ER stress and inflammatory response lead to massive apoptosis of cells. Our results showed that addition of PA had no significant effect on the mRNA levels of the inflammatory response-related genes (*IL-6*, *IL-1β*, and *IFN-γ*) and apoptosis-related genes (*Bax*, *Bcl-2*, *Caspase-3*, and *Caspase-9*) in goose primary hepatocytes, but in LO2 cells their mRNA levels markedly increased compared to the control group (p<0.05). Remarkably, the mRNA levels of Bax were increased more than 3-fold, which is consistent with the results in HepG2 cells [24]. Therefore, we conclude that goose hepatocytes were more tolerant to PA than LO2 cells in ER stress, inflammation, and apoptosis.

Results from previous studies reveled that overfeeding significantly increased expression of genes related to fat synthesis in the goose liver [13,25]. In the liver, PA elongation of the carbon chain is mainly catalyzed by ELOVL6 enzyme, and desaturation under the action of SCD1, FADS1, and FADS2, and finally forms non-toxic TGs under the action of DGAT2 [26]. And the above five genes mRNA levels significantly increased in the goose liver after overfeeding [13]. In our study, the levels of fatty acid desaturation-related genes (*SCD1* and *FADS2*) mRNA was markedly enhanced by addition of 0.6 mM of PA (p<0.05) in goose primary hepatocytes, while no significant differences were seen in LO2 cells. We also found that the expression of the fatty acid elongate enzyme-related gene (*ELOVL6*) in both cells significantly increased (p<0.05), whereas that of TG synthesis-related gene (*DGAT2*) remained statically unchanged in these two cells. Together, these results indicated that PA has an important effect on the fatty acid desaturation process in goose hepatocytes, and indicates that the fatty acid desaturation process may play an important role to PA tolerance of goose liver.

SCD1 is the key regulatory enzyme responsible for the desaturation of SFAs, which can desaturation PA into MUFAs. Moreover, increasing studies have shown that the mRNA levels of *SCD1* was increased in overfeeding-induced goose fatty liver [1,12,13]. Therefore, this study investigated the effects of *SCD1* on PA tolerance of goose primary hepatocytes. In our study, under the treatment of 0.6 mM PA, downregulation of *SCD1* decreased the tolerance of goose primary hepatocytes to PA (p<0.05), indicating that *SCD1* plays crucial role in PA tolerance of goose hepatocytes. Then, we further investigated the PA tolerance of goose primary hepatocytes in ER stress, inflammatory and apoptosis after downregulating with *SCD1*. Our data showed that downregulation of *SCD1* had no significant effect on the mRNA expression of ER stress-related genes (*XBP*, *BIP*, and *ATF6*), indicating that *SCD1* did not affect the PA tolerance of goose primary hepatocytes in ER stress response. Furthermore, *SCD1* downregulation resulted in the mRNA levels of inflammatory factors (*IL-6* and *IL-1β*) were significantly increased, suggesting that *SCD1* can regulate PA tolerance of goose primary hepatocytes in inflammation pathway where *IL-6* and *IL-1β* are located. Moreover, our results showed that downregulation of *SCD1*, the mRNA levels of apoptosis related genes (*Bax*, *Bcl-2*, *Caspase 3*, and *Caspase 9*) were not significantly changed, but the protein expression of cytochrome C and the apoptosis rate significantly increased, which indicated that *SCD1* increases PA tolerance goose primary hepatocytes by inhibiting apoptosis. These data demonstrated that *SCD1* plays a critical role in mediating PA tolerance of goose primary hepatocytes through inflammation and apoptosis response.

Finally, to further explore the specific pathways of *SCD1* regulating PA tolerance of goose primary hepatocytes, we tested the mRNA levels of key genes in the *SCD1* regulatory pathways. In our study, we found that under the treatment of 0.6 mM PA, downregulation of *SCD1* significantly increased the mRNA expression of *AKT1*, *AKT2*, *FOXO1*, and *SIRT1*, while that of *mTOR* and *AMPK* were not significantly altered, indicating *SCD1* can regulate the AKT/FoxO1 pathway at the transcription level. In addition, *SIRT1* can regulate *FoxO1* through acetylation, and early studies have demonstrated that *FoxO1* plays an important role in regulating inflammation and apoptosis [27–29], but the regulatory relationship between *SCD1* and *SIRT1* has not been reported. Therefore, we speculated that *SCD1* may regulate the AKT/FoxO1 pathway through SIRT1/FoxO1 pathway or increase the ratio of SFAs/MUFAs to inhibit the occurrence of inflammation and apoptosis response. Together, these data support a conclusion that *SCD1* may increase the PA tolerance of goose primary hepatocytes by regulating the AKT/FoxO1, SIRT1/FoxO1 pathways to inhibit the occurrence of inflammatory and apoptosis.

In summary, data from the present study suggested that goose primary hepatocytes have a higher tolerance to PA than LO2 cells and that *SCD1* has a crucial role in enhancing the PA tolerance of goose primary hepatocytes by regulating inflammation- and apoptosis-related genes expression.

## Figures and Tables

**Table 1:** The primers for quantitative real-time-polymerase chain reaction

Gene name	Forward (5′-3′)	Reverse (5′-3′)	TM (°C)
*g-β-actin*	CAACGAGCGGTTCAGGTGT	TGGAGTTGAAGGTGGTCTCG	60
*g-GAPDH*	GTGGTGCAAGAGGCATTGCTGAC	GCTGATGCTCCCATGTTCGTGAT	60
*g-SCD1*	GCCATCGGTCCTACAAAGC	AGCCAATGTGGGAGAAGAAA	60
*g-DGAT2*	CGCCATCATCATCGTGGT	CGTGCCGTAGAGCCAGTTT	60
*g-FADS2*	TCAACCATGATCAAGCGCAG	GACCCACACAAACCAGTGAC	60
*g-ELOVL6*	GCTAAGCAAAGCACCCGAAC	AGCCCGCAAGGCATAGTAAG	60
*g-BIP*	TGTAAATGGAATCCTCCGTGTCA	TCCGCAAACTTCTCAGCATCAT	60
*g-ATF6*	GGAGCACCACCGATTGACCTT	GGCTCAGGCTAACATTGGCATC	60
*g-XBP1*	AATGGGCAACCAAACCAATATG	GGCTGCAAGGCTACAAGGAGA	60
*g-IL-6*	AACTCTCCAGTGGGCTTTTC	TCACCATCTGCCTTATCGTC	60
*g-IFN-γ*	TGAGCCAGATTGTTTCCC	CAGGTCCACGAGGTCTTT	63.3
*g-IL-1β*	TCCGCCAGCCGCAAAGTG	CGCTCATCACGCAGGACA	61.4
*g-Caspase-3*	CTGGTATTGAGGCAGACAGTGG	CAGCACCCTACACAGAGACTGAA	60
*g-Caspase-9*	TTCCAGGCTCTGTCGGGTAA	GTCCAGCGTTTCCACATACCA	64
*g-Bax*	GAAGCATTTACAGTTGCCATTACAG	CCACAAGCAAGCAAAGAGCC	55
*g-Bcl2*	GATGCCTTCGTGGAGTTGTATG	GCTCCCACCAGAACCAAAC	60
*g-SIRT1*	CCTGCTCCCAGAAACAAT	GCTCCCGTCAAGACCATA	60
*g-FoxO1*	CATCCCTTCAGTCTGGTCAA	GAAAGGCTGGGTAAAGTAG	60
*g-AKT1*	TGCTGGATAAAGATGGAC	CTGGTTGTAGAAAGGGAG	60
*g-AKT2*	GCGATGCTCCATCACCTCC	CGCCTGCCCTTCTACAACC	60
*g-mTOR*	TCATTTGTTACTACCTCCCA	TTTCTAGAGCAGCTTTGCGAGCCAC	60
*g-AMPK*	TCGCCGTCTATCATCTCG	GTCCTACCTGCACAACCAA	60
*g-PI3K*	ACCCAAGCGAGGATGAGG	TGTTGCCCGTGTTGAATG	60
*h-β-actin*	TCATGAAGTGTGACGTGGACATCCGC	CTAGAAGCATTTGCGGTGGACGATG	60
*h-GAPDH*	AGAAGGCTGGGGCTCATTTG	AGGGGCCATCCACAGTCTTC	60
*h-SCD1*	TTCCTGGCTCTACCCTGTCTGTCC	GGGCACCCTCACCAAGTAAGC	60
*h-DGAT2*	GAGACTACTTTCCCATCCAG	GGTATCCAAAGATATAGTTCCT	60
*h-FADS2*	CCAGACTCCACTTCTCAA	GCCAGTTCACCAATCAGC	53
*h-ELOVL6*	ACAATGGACCTGTCAGCAAA	ATACCAGTGCAGGAAGAT	60
*h-BIP*	CGGGCAAAGATGTCAGGAAAG	TTCTGGACGGGCTTCATAGTAGAC	60
*h-ATF6*	GGAACAGGATTCCAGGAGAATGAACCCTAGTG	GATGTGTCCTGTGCCTCTTTAGCAGAAAATCC	60
*h-XBP1*	CAGAGTAGCAGCTCAGACTGCCAGAGATCG	GCTGTTCCAGCTCACTCATTCGAGCC	60
*h-IL-6*	CCTTGGGTCCAGTTGCCTTCT	CCAGTGCCTCTTTGCTGCTTTC	60
*h-IFN-γ*	CATCACGTCATACCAGCCATTT	CTGGATTGTCTTCGGTATGCAT	62
*h-IL-1β*	AGCCATGGCAGAAGTAC-CTG	TCCATGGCCACAACAACTGA	60
*h-Caspase-3*	GGAAGCGAATCAATGGACTC	CTCAGAAGCACACAAACAAAAC	60
*h-Caspase-9*	CATTTCATGGTGGAGGTGAAG	GGGAACTGCAGGTGGCTG	65
*h-Bax*	ATGTTTTCTGACGGCAACTTC	AGTCCAATGTCCAGCCCAT	55
*h-Bcl2*	CTTTGAGTTCGGTGGGGTCA	GGGCCGTACAGTTCCACAAA	60

g, h represents goose and human, respectively.

TM, temperature; *GAPDH*, glyceraldehyde-3-phosphate dehydrogenas; *SCD1*, stearyl-coenzyme A desaturase 1; *DGAT2*, diacylglycerol acyltransferase 2; *FADS2*, fatty acid desaturase 2; *ELOVL6*, elongase of very long chain fatty acids 6; *BIP*, binding immunoglobulin protein; *ATF6*, activating transcription factor 6; *XBP1*, x box-binding protein 1; *IL*, interleukin; *IFN*, interferon; *Bax*, bcl-2-associated X protein; *Bcl2*, b-cell lymphoma 2; *SIRT1*, sirtuin 1; *FoxO1*, forkhead box O1; *AKT1*, protein kinase B 1; *mTOR*, mammalian target of rapamycin; AMPK, adenosine 5′-monophosphate (AMP)-activated protein kinase; P13K, phosphoinositide 3-kinase.
